# Ivermectin – Old Drug, New Tricks?

**DOI:** 10.1016/j.pt.2017.02.004

**Published:** 2017-06

**Authors:** Roz Laing, Victoria Gillan, Eileen Devaney

**Affiliations:** 1 Institute of Biodiversity, Animal Health and Comparative Medicine, University of Glasgow, Garscube Estate, Glasgow G61 1QH, UK

## Abstract

Ivermectin is one of the most important drugs in veterinary and human medicine for the control of parasitic infection and was the joint focus of the 2015 Nobel Prize in Physiology or Medicine, some 35 years after its remarkable discovery. Although best described for its activity on glutamate-gated chloride channels in parasitic nematodes, understanding of its mode of action remains incomplete. In the field of veterinary medicine, resistance to ivermectin is now widespread, but the mechanisms underlying resistance are unresolved. Here we discuss the history of this versatile drug and its use in global health. Based on recent studies in a variety of systems, we question whether ivermectin could have additional modes of action on parasitic nematodes.

## Trends

Ligand-gated ion channels, particularly glutamate-gated chloride channels, are well characterised as the targets of IVM in nematodes and insects.

Nematode genomes are helping to cast light on the diversity of ion-channel subunits in different parasite species of human and veterinary importance.

Resistance to IVM is an increasing problem in the control of parasitic nematodes, and resolving the mechanisms is an important research priority.

Recent studies in other biological systems suggest that IVM can affect a number of additional pathways.

IVM may have novel applications in the treatment and control of important human diseases.

## From Golf Course to Nobel Prize

Ivermectin (IVM) is one of the best known and most widely used antiparasitic drugs in human and veterinary medicine. From a fortuitous discovery on a Japanese golf course to a Nobel Prize, the impact of IVM on human health to date has been extraordinary. Notwithstanding the role of IVM in global food production, the Mectizan Donation Program has lifted the burden of onchocerciasis (river blindness) and, subsequently, lymphatic filariasis (elephantiasis), from millions of people in the poorest countries in the world, and set a precedent for the role of public–private partnerships in global health. However, despite extensive research since its discovery over 35 years ago, the mode of action of IVM in parasitic species remains unclear, as are the mechanisms of resistance that allow some pathogens to survive treatment and thus the implications for current and future control strategies. Intriguingly, IVM has a diverse range of effects in many different organisms, far beyond the endoparasites and ectoparasites it was developed to control. For example, IVM has been shown to regulate glucose and cholesterol levels in diabetic mice [1], to suppress malignant cell proliferation in various cancers [2], to inhibit viral replication in several flaviviruses [3], and to reduce survival in major insect vectors of malaria and trypanosomiasis 4, 5. Clearly, much remains to be learned about this versatile drug, but the promise of more sustainable strategies for current helminth-control programmes and novel applications to improve and democratise human health, are compelling arguments to pursue this cause. In this article we review the current uses of IVM and discuss recent studies demonstrating a remarkably wide range of drug targets in different systems. We highlight some important but unresolved questions regarding drug mode of action and mechanism of resistance in parasitic nematodes, and suggest that recently available, high-quality genomic resources for parasitic helminths are the appropriate tools to answer to these longstanding questions.

## Discovery and Synthesis

In 1970, microbiologist Satoshi Ōmura collected a soil sample from woods close to a golf course in Kawana, on the south east coast of Honshu, Japan [6]. Ōmura isolated and cultured a Gram-positive bacterium, sample NRRL 8165–a then unknown species of *Streptomyces,* which was sent to William Campbell at Merck (along with 50 other strains of *Streptomyces* which were considered unusual in appearance or culture characteristics) to test for antiparasitic effects. NRRL 8165 cultures showed potent activity against *Nematospiroides dubius* (now known as *Heligomosoides polygyrus*) infection in mice, and the active components were purified, revealing a family of macrocyclic lactones. These naturally occurring compounds were named the avermectins (and the bacterium, *Streptomyces avermitilis*) to reflect the worm-free ‘averminous’ conditions they produced 7, 8.

**Figure 1:**
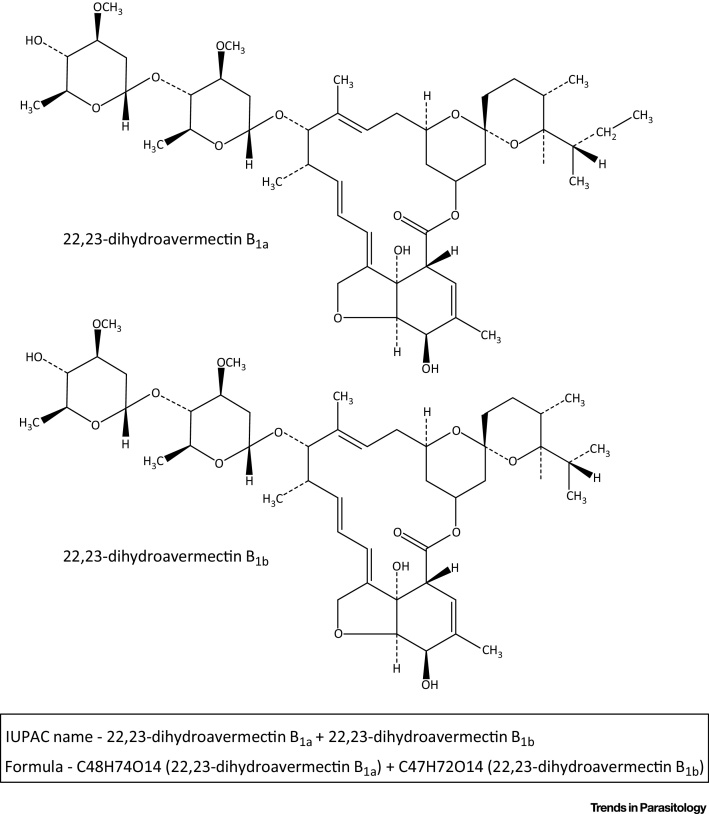
Chemical Structure of Ivermectin. Ivermectin consists of a mixture of two homologues: 5-O-dimethyl-22,23-dihydroavermectin B_1a_ and B_1b_ in a ratio of 80:20.

Naturally produced avermectins are a mixture of four compounds, avermectin A_1_, A_2_, B_1_, and B_2_, each of which exists as two variants, a and b 8, 9. The ‘A’ and ‘B’ designations describe the presence of methoxy or hydroxy groups at position C5, while the superscripts 1 and 2 refer to the presence of a double bond between C22 and C23 or a hydrogen at C22 and hydroxy group at C23, respectively. The ‘a’ variants have secbutyl at C25, while the ‘b’ variants have isopropyl. These subtle differences in chemical structure were found to have significant functional consequences; while initial trials found that all four avermectins showed some efficacy against gastrointestinal nematodes of sheep, avermectins of the ‘B’ series showed highest activity [10]. Further, when given orally, avermectin B_1_ was more active than B_2_, while with parenteral administration, avermectin B_2_ was more active than B_1_ [9]. On this basis, development of a commercial anthelmintic focused on the ‘B’ series and the chemical structure at the C22 and C23 positions. IVM is a chemically modified derivative of naturally produced avermectin B_1_, comprised of ∼80% 22,23-dihydro-avermectin B_1a_ and ∼20% 22,23-dihydro-avermectin B_1b_ 8, 9 (Figure 1), with potent activity against a broad spectrum of parasitic nematodes after both oral and parenteral administration. IVM is not active against flukes or tapeworms, but does have activity against various arthropods, including lice, mites, and some ticks. IVM has a wide safety margin in most mammals, although some dogs with a deletion mutation in MDR1, a P-glycoprotein that functions in the blood–brain barrier, are susceptible to neurological effects [11].

## Use in Veterinary and Human Medicine

The potency of IVM against both endoparasites and ectoparasites led to the creation of the term ‘endectocide’ and this first drug of its kind was introduced to the animal health market by Merck & Co. in 1981 [12]. New formulations of IVM for different livestock species and domestic pets were released almost every year, and, by the late 1980s, IVM was the largest selling animal health product in the world (http://merial.com/en/). A number of derivatives, such as eprinomectin (topical application for farm animals, with extended activity and no milk withdrawal [13]) and selamectin (topical application for small animals, with a wider safety margin than IVM in dogs with the MDR1 mutation [14]), have been developed since, to great commercial success. Two additional macrocylic lactones of commercial importance, moxidectin and milbemycin oxime, belong to a closely related but distinct family of *Streptomyces*-derived anthelmintics called the milbemycins. The key similarities and differences between the avermectins and milbemycins have been described elsewhere [15].

The market for IVM has remained exceptionally strong in the livestock industry, particularly for the control of gastrointestinal roundworms, although it is also licensed to control bovine lungworm and various ectoparasites. IVM and other macrocyclic lactones are currently the most commonly used anthelmintics in the UK sheep industry [16] and in the US cattle industry [17]. They are also the most frequently used anthelmintics to control equine roundworms in the UK 18, 19. Additionally, there is a large market for macrocyclic lactones in the control of parasitic nematodes and ectoparasites in domestic pets. IVM is licensed to control gastrointestinal roundworms (in combination with pyrantel in dogs) and the canine heartworm, *Dirofilaria immitis*. IVM is not active against the adult stages of *D. immitis,* but it is widely used to prevent disease by targeting the developing larvae following transmission from the mosquito. IVM is active during the first 6 weeks of infection, against the L3, L4, and juvenile adult, but does not risk the potentially catastrophic effects of dead and dying mature adult worms in the heart, which is key to its value in endemic areas [20].

While the potential value of IVM in the livestock and companion animal health market was recognised from the start, there was very little financial incentive to produce IVM for the human health market. However, its efficacy against the filarial nematodes responsible for onchocerciasis and lymphatic filariasis, moved Dr Roy Vagelos, CEO of Merck & Co., to donate as much IVM (licensed as Mectizan) ‘as was needed, for as long as needed, to anyone who needed it’ [21]. Since 1987, the Mectizan Donation Program has approved 1.4 billion treatments for the control and elimination of onchocerciasis, and 1.2 billion treatments (administered with albendazole, donated by GlaxoSmithKline) for the control and elimination of lymphatic filariasis (http://www.mectizan.org/resources/2014-annual-highlights). IVM does not kill adult *Onchocerca volvulus*, but a single oral dose (150 μg/kg) given annually suppresses microfilarial production and prevents disease progression [22]. Similarly, in lymphatic filariasis, IVM monotherapy does not kill adult *Wuchereria bancrofti* but is microfilaricidal, although in this case the suppression of microfilaria production is too brief to interrupt disease transmission 23, 24, 25. However, when IVM is administered annually with albendazole, control is highly successful [26]. Furthermore, recent studies found that a single dose of IVM administered with albendazole and diethylcarbamazine (DEC) resulted in complete clearance of microfilariae, which was maintained in all patients tested after 12 months (12 of 12 patients) and 24 months (6 of 6 patients) [27]. This compared to 1 of 12 patients who was microfilaria-free after a single dose of albendazole and DEC after 12 months. While the authors were unable to identify a pharmokinetic interaction between IVM and albendazole or DEC, the findings do suggest a novel synergistic effect, resulting in either permanent sterilisation or death of the adult stage of *W. bancrofti*. In addition to onchocerciasis and lymphatic filariasis, IVM also clears coinfection with a number of soil-transmitted helminths, including *Ascaris lumbricoides* and *Strongyloides stercoralis*, and some ectoparasites such as *Sarcoptes scabies* 28, 29, 30.

## Mode of Action

While the efficacy of IVM in treating a broad spectrum of parasitic infections is well established, its mode of action is less clear. At nanomolar concentrations, IVM affects nematode motility, feeding, and reproduction and acts via ligand-gated chloride channels, specifically those gated by glutamate 31, 32. Glutamate-gated chloride channels (GluCls) are not present in vertebrates, and as such are thought to confer the broad safety margin of IVM. However, at micromolar concentrations, IVM can interact with a wider range of ligand-gated channels found in both invertebrates and vertebrates, including GABA, glycine, histamine, and nicotinic acetylcholine receptors (reviewed in [33]).

GluCls are expressed in nematode motor neuron commissures, lateral and sublateral nerve cords, and pharyngeal neurons [34], and the effect of IVM on worm motility and feeding presumably relates to binding to GluCls at these sites [33]. Functional GluCls are composed of five subunits, with native GluCls containing multiple subunit types [33]. In the free-living nematode *Caenorhabditis elegans* there are six genes encoding GluCl subunits, of which *glc-1* is the major target of IVM 35, 36. However, the GluCl family appears to be remarkably divergent in parasitic nematodes, even in closely related species. The gastrointestinal parasite of sheep, *Haemonchus contortus,* and the human hookworms, *Necator americanus* and *Ancylostoma ceylanicum,* reside in the same phylogenetic clade as *C. elegans*, yet all lack *glc-1* orthologues 37, 38. Functional GluCl channels can, however, be generated from different combinations of subunits, and differences in the distribution and composition of the GluCl channels may contribute to differences in IVM susceptibility of different nematode species (strikingly, *A. ceylanicum* exhibits a 40- to 300-fold greater susceptibility to IVM than does *N. americanus* – *in vitro* and *in vivo* studies respectively 39, 40), as could differential sensitivity of the other ligand-channel types referred to above [33].

IVM also interferes with nematode fertility, a finding that is best characterised from studies on filarial worms, where it has long been recognised that IVM inhibited production of microfilariae *in utero* [41]. Transcriptomic analysis has since identified changes in gene expression following exposure of female *Brugia malayi* to 100 nM–1 μM IVM *in vitro* [42], with differentially expressed transcripts particularly enriched for those involved in female reproduction. Until recently, no GluCls had been reported in the nematode reproductive tract, so the effect of IVM on fecundity was thought to be indirect [33]. However, analysis of the *B. malayi* genome showed that a GluCl subunit, *avr-14*, was present [43], and using specific RNA probes this transcript was localised to the reproductive tract of adult *Brugia* [44]. *avr-14* was most strongly expressed in embryonic stages of microfilariae, as well as the uterine wall of the female worm and, to a lesser extent, the male reproductive tract, an observation that may help in defining the mechanism underlying IVM induced sterility.

As described in filarial nematodes, susceptibility to IVM can also vary between different life-stages of parasite, and there is growing evidence that interactions with the host immune response play a role in the activity of IVM. In *B. malayi* microfilariae, an antibody against a peptide derived from AVR-14-A was used to localise GluCl to the tissue surrounding the excretory–secretory (ES) apparatus only. IVM was proposed to cause a reduction in release of proteins from the ES vesicle, which may modulate host immune responses *in vivo* [43]. This hypothesis is consistent with findings in *D. immitis* microfilariae, where exposure to IVM *in vitro* resulted in increased binding of peripheral blood mononuclear cells and neutrophils [45]. Also, for both *D. immitis* and *O. volvulus* microfilariae, the *in vitro* effects of IVM required much higher concentrations than *in vivo* 45, 46, supporting a role for host immune function in the activity of IVM.

## Anthelmintic Resistance

IVM has been widely used in veterinary species for the prophylaxis and treatment of parasitic disease, often using a mass drug administration (MDA) strategy to protect all animals considered ‘at risk’. However, applying this blanket approach has resulted in rapid selection for parasitic nematodes that are capable of surviving drug treatment. Anthelmintic resistance is now a major global problem in the control of gastrointestinal roundworms of sheep, cattle, and horses [47], and there are now reports of IVM resistance in the canine heartworm, *D. immitis* [48]. Concurrently, reports of reduced embryostatic effects of IVM on *O. volvulus* in Ghana and Cameroon have raised concerns that IVM resistance may evolve in human parasites 49, 50, 51. In light of the rapid rise and spread of IVM resistance in the veterinary field, MDA of IVM as the sole means of control for onchocerciasis might be deemed a risky strategy, and there are calls for more integrated approaches [52]. While the potential impact of population structure and genetic diversity (with the potential bottleneck of vector transmission for the filarial nematodes) remain unclear, increased effort to develop sensitive markers of resistance is warranted.

In *C. elegans,* IVM resistance involves a number of genes. In 2000, Dent et al., found simultaneous mutation of three GluCl genes, *glc-1*, *avr-14*, and *avr-15*, conferred high levels of IVM resistance, with little or no resistance provided by mutations in any two of the genes [53]. The resistance phenotype was further modulated by mutations in the innexins, *unc-7* and *unc-9,* which are essential components of gap junctions and are required for normal locomotion and egg laying, and in four *dyf* genes, *osm-1*, *osm-5*, *dyf-11*, and *che-3,* which have roles in sensory neuron function [54]. A frameshift mutation in another *dyf* gene, *dyf-7*, has since been found to confer IVM resistance in two laboratory-selected isolates of *C. elegans* [54]. In natural populations of *C. elegans*, a four-amino-acid deletion in *glc-1* was found to confer abamectin and IVM resistance in multiple diverse populations, but other resistant populations lacked this mutation [36]. Further, in two out of six resistant populations with the *glc-1* mutation, the presence of a second dominant, but as yet unidentified, resistance locus was found [36].

For many years, these ‘candidate genes’ have been pursued in parasitic nematodes, particularly in *H. contortus* [55]. However, no robust association between any candidate gene and IVM resistance has been identified. In addition to the many studies investigating target-site mutations 56, 57, the roles of drug metabolism/excretion and drug uptake have also been a focus of much research (reviewed in [58]). These studies have correlated various polymorphisms or changes in expression of candidate genes with IVM resistance in particular isolates, but none have defined the major mechanism of resistance. To attempt to address this, a number of genome-wide sequencing and genetic crossing approaches are now being applied, facilitated by improvements in sequencing technologies and parasite genomic resources 38, 59, 60. Similar approaches have proven highly successful in determining the genetic basis of oxamniquine resistance in the human blood fluke, *Schistosoma mansoni* [61], and their application to parasitic nematodes is expected to rapidly improve our understanding of complex traits such as IVM resistance.

## New Targets and Novel Applications

**Figure 2:**
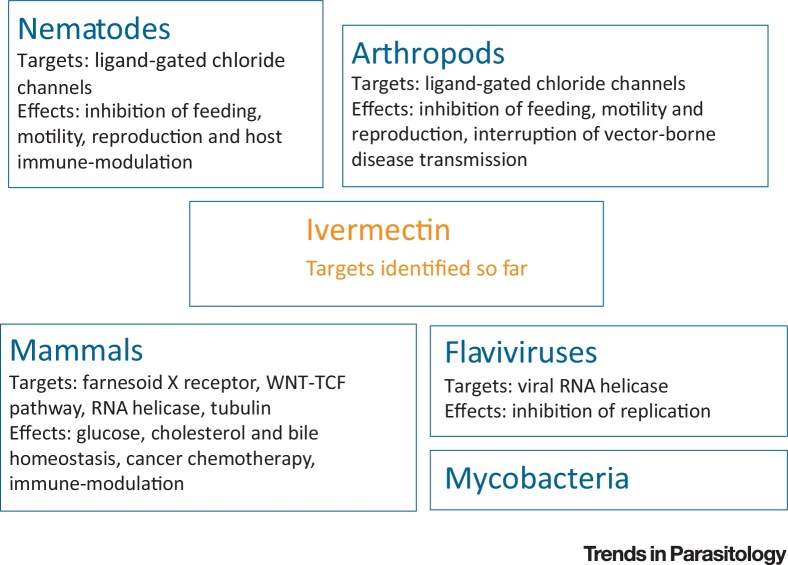
Key Figure: Ivermectin Targets Identified So Far

IVM is a potent anthelmintic with a wide safety margin, affecting susceptible nematodes when applied in nanomolar concentrations. However, at higher concentrations IVM has a broad range of effects in many different organisms (Figure 2, Key Figure, Table 1). Some of these effects may provide further clues to its mode of action in parasites and may have potential relevance in the treatment of human disease. A number of early studies discovered that, at high doses, IVM increases the chloride conductance of mammalian neuronal cells. On this basis, high-dose IVM (up to 1.6 mg/kg) has previously been used successfully for symptomatic treatment of severe muscle spasticity in patients with spinal cord injuries [62]. More recently, IVM was shown to induce intracellular chloride flux in human leukaemia cells *in vitro* [63]. This was associated with an increase in the production of intracellular reactive oxygen species (ROS), leading to cell death in leukaemia cells, but not in normal haematopoetic cells. This difference in susceptibility may reflect an increased expression of chloride channels on malignant cells or an increased susceptibility to ROS, both of which have been reported previously. IVM was also effective at slowing tumour growth *in vivo* in three mouse models of leukaemia [63], suggesting promise as a cancer chemotherapeutic.

**Table 1:** Additional Targets of IVM Reported from Other Systems

Organism/system	Predicted target	Dose (*in vivo*)	Concentration (*in vitro*)	Refs
Filarial nematodes (human)	Chloride channels	150 μg/kg	–	[22]
Gastrointestinal nematodes (sheep and cattle)	Chloride channels	200 μg/kg	–	8, 9
Leukaemia cells	Chloride channels	3–7 mg/kg	3 μM	[63]
Colon and lung tumour cells	WNT-TCF signalling	10 mg/kg	1–2.5 μM for concentration-dependent apoptosis; 1–2.4 μM IC50 antiproliferative activity	[64]
Glioma	RNA helicase DDX23	Intratumoural 3 mg/kg (50% decrease in tumour size), 10 mg/kg (near complete regression of tumour)	10 μM for downregulation of miR21; 25 μM for cell proliferation	[2]
HeLa cells	Tubulin	–	10 μM	[67]
Mouse	Farnesoid X receptor	1.3 mg/kg	–	[69]
T cells	Unknown	Topical 10 μl 0.1% IVM	1–3 mg/ml (1.14–3.42 mM)	[70]
Flaviviruses	RNA helicases	–	0.12–0.5 μM IC50 helicase activity; 0.019–0.354 μM for inhibition of helicase kinetics; 0.0005–4 μM to inhibit viral synthesis by 50%; 3.5–10 μM to reduce viability of cells for virus by 50%	[3]
*Mycobacterium* spp.	Unknown	–	MIC varied between 4 mg/L and >40 mg/L (4.57 μM and 45.70 μM)	72, 73

In addition to the effect of IVM on mammalian chloride channels, there is growing evidence that IVM may target alternative pathways relevant to cancer chemotherapy. In many human diseases, including cancers of the colon, skin, lung, breast, ovary, and prostate, there is deregulation of the WNT-TCF (WNT-T cell factor) signalling pathway (many WNT-TCF target genes regulate cell proliferation and metastasis). IVM has demonstrated *in vivo* efficacy against WNT-TCF-dependent human colon cancer and lung carcinoma xenografts, but not against WNT-TCF-independent tumours, suggesting specific blockade of the WNT-TCF response [64]. Yin et al. found that IVM also has *in vivo* efficacy against glioma xenografts, thought to function through inhibition of DEAD-box RNA helicase DDX23 [2]. This helicase is involved in the processing of a microRNA, *miR-21*, which is associated with glioma cell proliferation and invasion, and is overexpressed in many cancers. These findings may be linked, as microRNAs are known to regulate WNT-TCF pathways during development, as well as in various disease states [65]. Specifically, *miR-21* promotes colon cancer by directly inhibiting TGFβ-R2, which is a negative regulator of the WNT-TCF pathway [66]. More recently, *in vitro* studies have found that IVM has an antimitotic effect, via inhibition of microtubule depolymerisation [67].

In mammals, another major target of IVM appears to be the farnesoid X receptor (FXR), a nuclear hormone receptor involved in bile, cholesterol, and glucose homeostasis [68]. In diabetic mice, IVM reduces serum glucose and cholesterol levels and improves insulin sensitivity through activation of FXR, suggesting potential as a novel diabetic therapy. The closest nematode homologue to FXR is DAF-12, a nuclear hormone receptor which regulates development through life stages and determines adult lifespan in *C. elegans* [69]. Whether some of the effects of IVM on *C. elegans* could be attributed to binding to DAF-12 remains unresolved at present.

IVM has recently been shown to have anti-inflammatory properties in T cell-mediated skin disease, although the mechanism by which it exerts this effect is unknown [70]. Significant clinical improvement was achieved with IVM treatment in a murine model of atopic dermatitis, with a reduction in T cell activation, proliferation, and cytokine production. The effect does not appear to be mediated by FXR (which is also expressed by T cells) and the authors reported no interaction between IVM and other potential ligands expressed by T cells, such as the GABA type A receptor, despite a large-scale screening effort.

IVM has also shown promise in the treatment of certain viral pathogens. Consistent with the inhibition of RNA helicase DDX23 referred to above, IVM inhibits viral replication of several flaviviruses by blocking a viral helicase [3]. Susceptible flaviviruses include those causing yellow fever, dengue, West Nile virus and tick borne encephalitis, and a patent application has been submitted for off-label antiflavivirus therapy in humans (patent application EP2010/065880). Encouragingly, serial passage of yellow fever virus with increasing concentrations of IVM did not appear to select for viral resistance, even after more than 30 passages over 6 months, leading the authors to conclude that adaptive mutations in the helicase domain may not be viable. In that study, no antiviral effect was detected in other genera of viruses, but inhibition of HIV-1 (and dengue) replication was reported after *in vitro* exposure to high concentrations (25–50 μM) of IVM. In this case, suppression of viral replication was thought to reflect disruption of viral protein trafficking between the host cell cytoplasm and nucleus by IVM inhibition of importin α/β-mediated transport [71].

Although IVM has a similar chemical structure to the macrolide antibiotics, it lacks activity against most bacteria. However, a number of studies have investigated IVM as an antimycobacterial agent, with varying degrees of success. One report described promising *in vitro* activity of IVM against various species of *Mycobacterium,* including *Mycobacterium tuberculosis*, the causative agent of tuberculosis [72]. However, a second study highlighted significant differences in the concentrations of IVM that were required to inhibit growth of the same *Mycobacterium* spp. and revealed a broad spectrum of susceptibility in clinical *M. tuberculosis* isolates [73]. More recently, *in vitro* activity of IVM against *Mycobacterium ulcerans,* the causative agent of Buruli ulcer, has been described [74]. In all studies, the *in vitro* antimycobacterial effects of IVM required significantly higher concentrations of drug than for antiparasitic effects, but the dosage for *in vivo* efficacy remains to be established. The mode of action is unknown.

IVM has well established efficacy against a wide range of arthropods and may have potential in breaking transmission of human disease through vector control. IVM given to local cattle has been shown to reduce the survival and fecundity of the tsetse fly *Glossina palpalis gambiensis* that transmits animal and human trypanosomiasis (sleeping sickness) in sub-Saharan Africa [5]. Many farmers already use IVM to control gastrointestinal parasites in their cattle, so this could form part of an integrated control strategy. Similarly, IVM ingested with host blood (modelled on plasma concentrations after a standard oral dose of 150 μg/kg) was found to reduce survival and blood feeding of *Anopheles gambiae,* the mosquito that transmits *Plasmodium falciparum* malaria [75]. The authors of that study suggested that an increased frequency of IVM treatment could break the cycle of malaria transmission, but highlighted the importance of field conditions and the potential development of resistance. Interestingly, different mosquito species are not uniformly susceptible to IVM. *Aedes aegypti,* the mosquito vector of yellow fever, dengue, and Zika, was not affected at IVM concentrations relevant to those in human blood following standard dosage, although survival and fecundity was affected at higher doses 75, 76. Furthermore, different strains of *A. aegypti* varied by approximately threefold in their adult survival rate after IVM ingestion, although the egg hatch rate varied to a lesser extent between strains and was not correlated to adult survival rate. This finding led the authors to speculate that IVM might affect mosquito survival and fecundity through different pathways [76].

## Concluding Remarks

Despite over 30 years of use in veterinary species, and nearly 30 years use in human medicine, there is much to learn about IVM (see Outstanding Questions). The precise mode of action in helminth parasites is still unknown, but the relationship between host immunity and drug efficacy is intriguing and worthy of further study. Similarly, the mechanisms underlying IVM resistance are unclear, and determining the genetic basis of resistance remains a pressing issue. However, the availability of multiple parasite genomes for comparative analysis, and the application of high-throughput sequencing technologies to classical genetic approaches, may provide answers to these questions soon. While IVM has already lifted the burden of onchocerciasis and lymphatic filariasis from millions of people, it is also likely that IVM (or novel derivatives) may prove valuable in the treatment of other important diseases. Further, the incredibly broad range of effects of IVM, in a wide variety of systems, may offer new insights into its mode of action in the original target species – the parasitic worm.

Outstanding Questions While ligand-gated ion channels are clearly a target of IVM in nematodes, are there additional targets, perhaps in distinct tissues of the worm?

IVM induces various phenotypes in susceptible nematodes, including paralysis, inhibition of feeding and reproduction. Are these phenotypes all dependent upon the same mode of action?

How conserved is the mode of action of IVM in different nematode species? What underlies the large differences in IVM susceptibility in closely related species (e.g., hookworms, mosquitoes)?

What is the relationship between IVM therapy and the immune response? Is the hypothesis of a host immune component in microfilariacidal activity the exception or the rule? What is the target in T cell suppression?

How is IVM resistance manifest at a molecular level in parasitic nematodes? What are the mechanisms, and are they conserved between (or even within) species?
